# Resilience Significantly Contributes to Exceptional Longevity

**DOI:** 10.1155/2010/525693

**Published:** 2010-12-06

**Authors:** Yi Zeng, Ke Shen

**Affiliations:** ^1^Center for the Study of Aging and Human Development and Geriatric Division of Medical School, Duke University, Box 3003, Room 1506, BUSSE Building, Durham, NC 27710, USA; ^2^China Center for Economic Research, National School of Development at Peking University, Beijing 10087, China; ^3^Max Planck Institute for Demographic Research, Konrad Zuse Straße 1, 18057 Rostock, Germany

## Abstract

*Objective*. We aim to investigate whether centenarians are significantly more resilient than younger elders and whether resilience significantly contributes to exceptional longevity. *Data*. We use a unique dataset from the Chinese Longitudinal Healthy Longevity Survey with the largest sample to date of centenarians, nonagenarians, octogenarians, and a compatible group of young old aged 65–79. *Methods and Results*. Logistic regressions based on the cross-sectional sample show that after controlling for various confounders, including physical health and cognitive status, centenarians are significantly more resilient than any other old-age group. Logistic regression analyses based on the longitudinal data show that nonagenarians aged 94–98 with better resilience have a 43.1% higher likelihood of becoming a centenarian compared to nonagenarians with lower resilience. *Conclusions*. Resilience significantly contributes to longevity at all ages, and it becomes even more profound at very advanced ages. These findings indicate that policies and programs to promote resilience would have long-term and positive effects on the well-being and longevity for senior citizens and their families.

## 1. Introduction

Extant literature has highlighted the particular relevance of centenarians for healthy longevity research, given that they have outlived most of their cohort peers by several decades, and they represent a highly selected group. For example, according to recent life-table data, the probability of surviving from age 50 to age 100 is about 0.38% and 1.05% for Chinese men and women, respectively, which is slightly less than one-fifth of that in the U.S. As shown by Flachsbart [1], only about 4.8% of 90-year-olds and 16.0% of 95-year-olds in Germany are likely to reach the age of 100. A study by Yi and Vaupel [2] showed that about 3.4% of 90-year-olds and 14.9% of 95-year-olds in China are likely to survive to the age of 100. These figures indicate that centenarians are highly selected long-lived individuals, even among those who have reached 90–95 years old. A focus on extreme cases is often a good way to gain research leverage at reasonable expense; thus, investigating centenarians (some of whom are healthy and demonstrate successful aging) and comparing them with other younger age groups is an efficient way to learn what factors may contribute to healthy longevity. 

Resilience, a psychological construct, has been defined differently in extant literatures. In this paper, we adopt the simplified and straightforward definition specified by Lamond et al. [3]; namely, resilience connotes the ability to adapt positively to adversity. Previous studies have demonstrated that resilience is generally positively correlated with cognitive function, physical health and self-reported health among the elderly [4–6], as well as with self-rated successful aging [3] in developed countries. Poon [7] discovered that the common characteristics of the Georgia centenarians sample are optimism and flexibility, which are documented to be associated with resilience [8]. Based on a Swedish sample, Nygren [9] found that mean resilience scores are higher in their oldest old sample (over age 85) compared to the scores of the younger adults. Selim [10] discovered that U.S. centenarian veterans are psychologically resilient despite their poor physical health. Jopp and Rott [11] also demonstrated that psychological resilience is well preserved at the very end of the life span based on the Heidelberg Centenarian Study. 

However, there are three major limitations in the previous research on centenarians and resilience. First, most of the prior studies were based on small samples with limited numbers of centenarians and nonagenarians, which restricted estimation efficiency [11, 12]. After a careful literature search, we have discovered that among all published studies concerning the characteristics and effects of resilience among centenarians with a comparison to other age groups, the largest sample was 272 Japanese centenarians by Yukie et al. [13]. 

Second, though some studies compared resilience between centenarians and young-old groups [7, 8, 10, 11], no prior research (to our knowledge) has explored whether resilience contributes to exceptional longevity at very advanced ages 95+.

Third, almost all previously published studies in this field dealt with developed countries; we found very few studies on centenarians and resilience from developing countries [2, 14, 15]. Shen and Zeng [15] reported on the positive association between resilience and survival among the Chinese elderly aged 65+, but they did not investigate whether the association still held at extremely advanced ages, for example, age 95 and older. As Ju and Jones [16] noted, in high-mortality populations, the oldest old are those highly selected individuals who have survived dangers when being born, risks in infancy and childhood, and hunger, sickness, and accidents at middle- and young-old ages. As evidence of the high-mortality selection in China, there were about five centenarians per million in China in the 1990s, compared with 50 per million in Western Europe [17]. Another important factor is that facilities to assist oldest old persons in their daily life are less likely to be available in developing countries than in industrial countries. This may force the oldest old in developing countries to perform daily activities by themselves, and the frequent exercise may enable them to maintain their physical capacities for a longer time than their counterparts in developed countries. These factors may help to explain why the elderly in Indonesia, Malaysia, the Philippines, Singapore, and Thailand have, in some comparative studies, found to be more active than the elderly in developed countries [16, 18]. Similarly, the functional capacity of centenarians in the three cities of Beijing, Hangzhou, and Chengdu in China has also been reported to be significantly better than that of Danish centenarians [19]. Thus, research on centenarians from developing countries including China, where the oldest old individuals are highly selected from poor early-life conditions and severe adversities, may be useful for identifying what factors may affect exceptional longevity. 

Based on the cross-sectional data from 2008-09 wave of the Chinese Longitudinal Healthy Longevity Survey (CLHLS) consisting of 16,566 elderly aged 65+ with 3,413 centenarians and 4,596 nonagenarians, as well as the follow-up data from the exceptionally long-lived individuals in the CLHLS 2002, 2005, and 2008-2009 waves, we test the following hypotheses.

H_1_ after controlling for various potential confounders including physical health and cognitive status, centenarians are significantly more resilient than the younger elderly.
H_2_ better resilience significantly contributes to exceptional longevity at very advanced ages.

## 2. Data, Measurements, and Methods

### 2.1. Data Source

The datasets used in this paper are from the CLHLS, which has been conducted in 1998, 2000, 2002, 2005, and 2008-2009 in a randomly selected half of the counties/cities in 22 Chinese provinces, covering 85% of the total population in China [20]. The 1998 baseline and the 2000 followup surveys interviewed the oldest old aged 80 and above only; since the 2002 wave, younger-old respondents aged 65–79 were also included in the sample. Careful and systematic evaluations (such as reliability coefficients and factor analysis, etc.) have shown that the data quality of the CLHLS surveys is reasonably good [21]. 

Cross-sectional data from the newest CLHLS wave conducted in 2008-2009, with a total sample size of 16,566 elderly aged 65 and above including 3,413 centenarians and 4,596 nonagenarians, is used to test hypothesis H_1_.  Table 1 presents the sample distribution of the CLHLS 2008-2009 wave by urban-rural residence, age groups, and gender. 

Comparative analysis on the resilience scores between centenarians and the other age groups may only reveal the “de facto” differences in resilience scores between the centenarians and the younger elders. Such cross-sectional analysis may not be suitable to examine the impact of resilience on attaining exceptional longevity as proposed by hypothesis H_2_. Thus, we conduct another set of multivariate analyses based on the follow-up data including those aged 94–97 in the 2002 survey and those aged 97-98 in the 2005 survey (we include the nonagenarians who were interviewed in the 2002 and 2005 waves rather than the 1998 baseline and 2000 wave of CLHLS, because two of the seven resilience questions were not asked in the 1998 baseline and the 2000 wave). The rationale for these analyses is that we wish to investigate whether resilience significantly contributes to surviving to 100 years old before or in the 2008-2009 interview among the nonagenarians who are the potential candidates and need to survive for at least two more years to reach age 100. The eligible sample consists of 585 men (38.3%) and 943 women (61.7%), all of whom were either interviewed at age 94–97 in 2002 or interviewed at age 97-98 in 2005. Among them, 1,049 (68.7%) died at ages <100 (failed to become a centenarian) and 479 (31.3%) survived to age ≥100 before or in the 2008-2009 survey (successful in becoming a centenarian). 

### 2.2. Measurements

#### 2.2.1. Variables of Interest

**Resilience**

As the datasets used in this paper are derived from the CLHLS which is a typical demographic study on determinants of healthy longevity rather than a detailed psychological investigation, we use a simplified resilience score (abbreviated as SRS hereafter) emphasizing coping and adjustment among the elderly. Our SRS is theoretically guided by the general framework of, but different from, the Connor-Davidson Resilience Scale (CD-RISC) [22]. The SRS is based on the available data collected through seven questions related to resilience in the CLHLS (See Table 2). In general, the seven items reflect personal tenacity, optimism, coping with negative mood, secure relationship, and self-control, which are deemed as important factors of resilience [22]. These items have their own counterparts in CD-RISC which deliver similar meanings. For example, item 1 of SRS corresponds to the item “Think of self as strong person” in CD-RISC; item 2 corresponds to the item “See the humorous side of things”; items 3 and 4 correspond to the item “Can handle unpleasant feelings”. In fact, some previous studies have also proposed resilience scales with very limited number of items focusing on one or two specific aspects of resilience. For example, Smith [23] proposed a Brief Resilience Scale (BRS), which included 6 items and focused on the ability to bounce back or recover from stress. Sinclair and Wallston [24] constructed a Brief Resilient Coping Scale (BRCS) with 4 items, emphasizing on the resilient coping process.

Items 1, 2, 3, 4, and 7 of SRS in the CLHLS carry a five-point (0, 1, 2, 3, and 4) range of the responses (see Table 2). We dichotomize the scores of items 5 and 6, because the rather detailed multiple choices of the answers for these two items only allow us to do so. With such scores which contain the maximum information, we can obtain from the survey the total SRS ranges from 0 to 22, with higher scores reflecting greater resilience.

The internal consistency of SRS measured by Cronbach's alpha coefficient is 0.69, indicating its reliability is reasonably adequate [25]. Principle component analysis generates three factors with eigenvalues >1, explaining 78.5% of the total variance. These basic indicators of the psychometric properties show that the SRS based on the CLHLS data are reasonably acceptable.

If the respondents are able to answer the questions 5 and 6 regarding social support, they do so; otherwise, proxy responses are allowed because spouses and close relatives typically know respondents' sources of social support (proxy responses are widely used in interviews with elderly [26]. The proportions of proxy answers for question 5 and 6 in the CLHLS are 27.17% and 26.97%, respectively. We also conducted regression analyses excluding the cases with proxy responses, and found very similar results between excluding and including the proxy responses). Questions concerning resilience items 1–4 and item 7 (see Table 2) relate to the self-feelings of the elderly and may not be judged accurately by others; thus, they are required to be answered by the elderly respondent him/herself in the survey. As a result, 17.1% of the respondents are unable to answer these questions due to poor cognitive ability. Simply, excluding these cases with the missing values might lead to sampling bias. Thus, we conduct multiple imputations for the missing values of these five resilience items based on the respondents' age, gender, race, education, physical health measured by Activities of Daily Living (ADL), and cognitive status measured by Mini Mental State Examination (MMSE) [27].

#### 2.2.2. Potential Confounding Factors

**Age Group**

We categorize the continuous age variable into 4 groups: ages 65–79 (reference), ages 80–89, ages 90–99, and ages ≥100.

**Demographic and Socioeconomic Characteristics**

We include gender (male or female), race (Han ethnicity or minority), residential place (urban or rural), marital status (currently married or unmarried including widowed, divorced, and never married), education (literate or illiterate), and pension status (with or without pension) as demographic and socioeconomic controls. Each control variable is measured as a dummy variable.

**Health Status**

Health status is measured by ADL and MMSE, which are based on international standards and adapted to the Chinese culture and social context with carefully conducted pilot study tests. More specifically, ADL is based on Katz's ADL index, representing the physical health of the elderly [28]. If a person needs help to perform any of the six daily tasks of bathing, dressing, indoor transferring, going to the toilet and cleaning oneself afterwards, eating, and continence, he/she is considered to be ADL dependent; otherwise, he/she is regarded as ADL independent. MMSE has been widely used for assessing cognitive mental status both in clinical practice and in research [29, 30]. The MMSE questionnaire includes 24 items with a total possible score of 30. We define those who have a score of less than 24 as cognitively impaired [30].

Resilience might have an indirect impact on survival through health status of the elderly. Thus, we control for health status in the models so that we can examine whether resilience has a direct impact on survival among the elderly. A similar design has been used in previous studies. For example, to examine the effect of religious attendance on mortality, Hill et al. [31] first ran the regression without controlling for health status and then included ADL, cognitive function, and other health measures in the model to see whether the effect of religious attendance on survival persisted and whether part of the effect of religious attendance was mediated through health status.

### 2.3. Methods

In the empirical analyses, the original SRS variable (ranging from score 0 to 22) is dichotomized into two categories according to the mean of resilience scores: higher resilience (with a resilience score ≥16) and lower resilience (with a resilience score <16). We apply logistic regression based on the cross-sectional data from the CLHLS 2008-2009 wave to test hypothesis H_1_ concerning whether centenarians are more resilient than any other age group. The independent variables of main interest are three age group dummy variables: centenarians, nonagenarians, and octogenarians, with age group 65–79 as the reference category. Potentially confounding variables including demographic and socioeconomic characteristics and health status are outlined above.

To test hypothesis H_2 _“Better resilience significantly contributes to exceptional longevity at very advanced ages,” we apply the multivariate logistic regression model based on the longitudinal data. The dependent variable of the logistic regression is whether the nonagenarian aged 94–97 in 2002 or aged 97–98 in 2005 survived to age ≥100 or died at age <100 before the CLHLS 2008-2009 survey. The key independent variable is whether the elderly enjoy higher resilience (with an SRS ≥16) or lower resilience (with an SRS <16). We control for age, gender, race, residence, marital status, and pension status as well as physical health and cognitive status measured by ADL and MMSE. We also adjust for the year of the interview and for dummy variables indicating province.

## 3. Results

### 3.1. Descriptive Analysis

As shown by the descriptive statistics based on the cross-sectional data presented in Table 3, the mean SRS are the highest for the young-old aged 65–79 without controlling for other covariates, while there are very little differences among the octogenarians, nonagenarians, and centenarians. Men have better SRS than women. A much larger proportion of the older men are literate and enjoy pension compared to the older women, due to the social background of gender inequality in China in the past. Additionally, men are more advantaged in ADL and MMSE than women in each of the age groups. 

**Figure 1:**
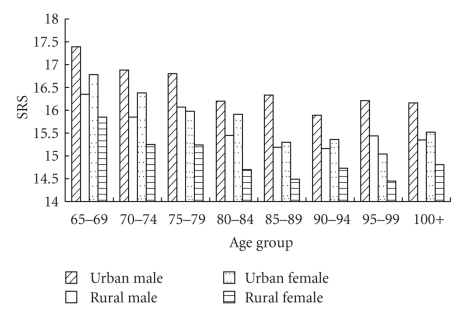
Average SRS by age groups, gender, and urban-rural residence.

Figure 1 depicts the weighted average SRS by 5-year age groups, gender, and urban-rural residence. Elderly men are always more resilient than their female counterparts in each of the age groups depicted in the figure. In general, the average SRS in urban and rural among the young old aged 65–69 is the highest and then decline as age increases. However, the SRS remains relatively stable after age 85. The Chinese elderly residing in urban area have higher resilience scores than their rural counterparts (see Figure 1). 

### 3.2. Centenarians Are Significantly More Resilient than Any Other Elderly Adults, After Controlling for Various Confounders Including Health

Table 3 and Figure 1 have shown that the average SRS of centenarians is lower than that of the younger respondents aged below 85 (except the average SRS of the female rural centenarians which is slightly higher than that of female rural elderly aged 80–84).  Table 4 presents estimates of the odds ratios regarding the associations of age-group dummy variables with better resilience among the elderly population. The results of model 1 indicate that without adjusting for any confounders, the likelihoods of enjoying better resilience for the octogenarians, nonagenarians, and centenarians are 40.3%, 55.7%, and 69.7% (*P* < .01) lower than that for the young old aged 65–79. After adding demographic and socioeconomic characteristics into model 2, the odds ratios for the octogenarians, nonagenarians, and centenarians (as compared to the young old aged 65–79) increase substantially, but are still significantly less than 1.0, indicating that the age difference is largely reduced with the addition of these controls. Model 3 further adjusts for physical health and cognitive mental status measured by ADL and MMSE; once these health variables are controlled for, centenarians are in fact, significantly more resilient than the young old by a margin of 26.1% (*P* < .01), nonagenarians also marginally enjoy better resilience than the young old by a margin of 10.9% (*P* < .1), while there is no significant difference in resilience between octogenarians and the young old (we also ran ordinary least square regressions using the original SRS scores (ranging from 0 to 22) as the continuous dependent variable, and the results (data not shown due to space limitations) support the same conclusion as the logistic regressions do shown in Table 4). These results show that centenarians are a highly selected and special subpopulation with the best resilience after controlling for various confounders including health. 

We further estimate the odds ratios separately for men and women in Model 4 and Model 5. We find that after adjusting for all potential confounders including ADL and MMSE, male and female centenarians are significantly more resilient than the young old, and the difference is substantially larger among men than among women. 

 The age pattern of resilience presented in Table 4 is, in general, similar to the findings from the study by Nygren and colleagues [9] on a Swedish oldest old sample aged 85 and older and the research by Lamond and colleagues [3] on the American elderly aged 65 and older, though both of these two studies did not provide explicit estimates for centenarians. We discover that centenarians' average SRS without adjusting for confounders is lower than that of the young-old due to their poorer objective health. After the health status variables are controlled for, the centenarians are much more resilient than the young old.

Regarding the confounding variables, we find that after adjusting for various other confounders, men are significantly more resilient than women; the elderly residing in urban areas, having at least one year of schooling, and enjoying pension benefits have significantly better resilience. Marriage has a very strong positive impact on resilience. Better physical health and cognitive status significantly improve resilience (see Table 4).

### 3.3. Better Resilience Significantly Contributes to Exceptional Longevity at Very Advanced Ages

As shown in Table 5, the likelihood of surviving to age 100 for the elderly aged 94–97 in 2002 or aged 97-98 in 2005 with higher resilience score was 74.6% (*P* < .01) higher than the likelihood of their counterparts with lower resilience scores in Model 1 with no controls. After the demographic and socioeconomic characteristics are adjusted for in Model 2, the odds ratio for high resilience slightly increase to 1.877, which indicates that the nonagenarians aged 94–97 with a higher resilience score have a 87.7% (*P* < .01) higher chance of becoming a centenarian as compared to their peers with similar demographic and socioeconomic characteristics but a lower resilience score. After further adjusting for health outcomes, the odds ratio substantially reduces from 1.877 to 1.431, but remains highly significant (*P* < .01). This indicates that resilience has a direct effect on achieving exceptional longevity, while part of its total effect operates indirectly through the pathway of health. We also further estimate the odds ratios separately for men and women in Models 4 and 5, controlling for demographic and socioeconomic characteristics and health outcomes. It turns out that the male and female nonagenarians with higher resilience scores have about 51.5% (*P* < .1) and 40.4% (*P* < .05) higher chances to become a centenarian, respectively. 

 To our knowledge, there are no published studies which have investigated the association of resilience with surviving to age 100+ among nonagenarians. However, the findings of our present study can be explained by the general literature on resilience as the ability to adapt positively to adversity among old adults [3]. When individuals reach very advanced ages, accumulated negative conditions such as health deterioration and bereavement of loved family members represent serious challenges. Thus, nonagenarians who are more resilient may have stronger capacities and potentials for dealing successfully with these serious challenges, constraints, and adversities [11] to subsequently survive to age 100+.

## 4. Discussions and Conclusion

Based on a cross-sectional dataset with the largest sample size to date of centenarians, nonagenarians, and octogenarians plus a compatible group of young-old aged 65–79, the descriptive statistics show that the average resilience score among centenarians is lower than that of the other elderly age groups. However, our multivariate logistic analyses based on the same dataset have confirmed the hypothesis that after controlling for various potential confounders including physical health and cognitive status, centenarians are significantly more resilient than any other age group of the elders. 

We also investigate the role resilience played in contributing to exceptional longevity based on the follow-up (up to 2008-2009) data from nonagenarians aged 94–97 in 2002 or aged 97-98 in 2005. The results confirm that better resilience contributes significantly to the likelihood of becoming a centenarian (i.e., exceptional longevity) among the nonagenarians. 

A previous study based on CLHLS longitudinal data showed that on average, better resilience reduced mortality risk by about 15.5% among all elderly adults aged 65+, adjusted for various confounding factors including physical and mental health [15]. Our present multivariate analysis focuses on centenarians and nonagenarians. We have shown that the positive effect of the resilience on enhancing the likelihood of surviving to the age of 100 among nonagenarians aged 94–98 is 43.1% (*P* < .01) for both genders combined (51.5% (*P* < .1) for men and 40.4% (*P* < .05) for women). It seems that the positive effects of the resilience on survival at very advanced ages may be more profound. 

Why is resilience positively associated with survival at very advanced ages in China? One possible explanation is that resilience is correlated with improved physical and psychological health, and better health increases the probability of survival. As shown in our logistic regression analyses, the effect of resilience on the likelihood of becoming a centenarian shrinks substantially after variables of initial health status are included in the model. Prior investigations also lend support to this explanation. For instance, Wagnild [5] and Lamond [3] indicated that resilience was positively associated with physical and cognitive function. Ong [32] demonstrated that resilient individuals were more likely to hold positive emotions, which promoted both resistance to and recovery from stress and thus contributed to better health and longevity. 

While the study presented in this paper is unique in terms of its largest sample size of centenarians and nonagenarians and interesting findings about the remarkable and significant effect of resilience on exceptional longevity at very advanced ages in a developing country, it should be interpreted with caution due to its inherent limitations. First, because the CLHLS is a demographic survey focusing on the determinants of healthy longevity such as demographic characteristics, socioeconomic status, life style, and health status of the elderly, we cannot assess as many resilience indicators as with other specific psychological surveys. Future research that collects more sophisticated psychological data could deepen our understanding of the impacts of resilience on healthy longevity in China. Second, we only examine the impact of resilience on exceptional longevity at advanced ages, rather than the mechanisms of how resilience works. Perhaps resilience strengthens the positive function of the immune system and certain gene(s) or ameliorates the negative impacts of some other gene(s). More detailed phenotypic and genotypic data and advanced interdisciplinary research across social and biomedical sciences are called for to explore the mechanisms.

In conclusion, the present study provides strong evidence to support the hypotheses that after adjusting for various confounders including current health, centenarians are significantly more resilient than any other age group of the elderly population and that better resilience contributes significantly to exceptional longevity even at very advanced ages. These findings are not only scientifically meaningful but also have policy relevance, indicating that policies and programs aiming at the promotion of resilience among old citizens ought to be put on the agenda. We may learn from the successful practices in other countries, such as resilience-promotion interventions conducted in Norway, which involve promoting expression of feeling and encouraging participants' attempts to make meaningful connections between their past, present, and future lives [33]. Such efforts would have long-term and positive effects on the well-being and healthy longevity for elderly citizens and their families.

## Figures and Tables

**Table 1:** Sample distributions of the elderly respondents in the CLHLS 2008-2009 wave.

Age group	Urban			Rural			Rural-urban combined		
	Men	Women	Total	Men	Women	Total	Men	Women	Total
65 –69	277	295	572	462	372	834	739	667	1,406
70 –79	605	560	1,165	913	801	1,714	1,518	1,361	2,879
80 –89	825	829	1,654	1,323	1,295	2,618	2,148	2,124	4,272
90 –99	817	1,075	1,892	1,080	1,624	2,704	1,897	2,699	4,596
100+	319	974	1,293	369	1,751	2,120	688	2,725	3,413
Total	2,843	3,733	6,576	4,147	5,843	9,990	6,990	9,576	16,566

**Table 2:** Measures of resilience: questions of the seven items asked in the CLHLS interviews.

Items	Item statements questions	Scores based on answers
Item 1	Do you feel the older you get, the more useless you are?	0. always; 1. often; 2. sometimes; 3. seldom; 4. never
Item 2	Do you always look on the bright side of things?	4. always; 3. often; 2. sometimes; 1.seldom; 0. never
Item 3	Do you often feel fearful or anxious?	0. always; 1. often; 2. sometimes; 3. seldom; 4. never
Item 4	Do you often feel lonely and isolated?	0. always; 1. often; 2. sometimes; 3. seldom; 4. never
Item 5	To whom do you usually talk most frequently in daily life?	1. Family members/friends/neighbors/social workers/caregivers; 0. Nobody.
Item 6	Who do you ask first for help when you have problems/difficulties?	1. Family members/friends/neighbors/social workers/caregivers; 0. Nobody.
Item 7	Can you make your own decisions concerning your personal affairs?	4. always; 3. often; 2. sometimes; 1.seldom; 0. never

**Table 3:** Descriptive statistics of the variables investigated in this study.

	Ages 100+		Ages 90–99		Ages 80–89		Ages 65–79	
	Men	Women	Men	Women	Men	Women
Mean SRS	15.8	15.0	15.6	14.9	15.8	14.9	16.6	15.7
Mean age	101.9	102.4	93.1	93.6	84.6	84.8	71.9	72.0
% Han ethnicity	93.6	93.4	94.6	93.2	94.6	94.1	94.0	94.1
% urban residence	45.4	35.0	43.0	39.7	38.3	39.0	39.2	42.2
% currently married	11.3	1.1	24.8	5.0	47.7	18.5	77.0	52.2
% literate	43.2	6.6	54.0	12.6	60.6	16.6	78.5	39.5
% having pension	23.6	2.7	27.9	7.3	27.3	9.2	33.2	21.5
% ADL independent	52.0	46.9	76.2	70.2	89.2	87.2	96.5	96.0
% normal cognition	33.3	16.6	49.3	32.4	72.2	57.2	92.1	85.9
Subsample size	666	2,677	1,883	2,677	2,139	2,110	2,253	2,023

Note: the mean SRS among the elderly aged 65–79, 80–89, and 90–99, presented in this table and in Figures 1(a) and 1(b) are weighted averages, using the 2000 census rural-urban-sex-age distributions and the corresponding CLHLS 2008-2009 sample distributions to compute the weights. The mean resilience scores of the centenarians are unweighted as the CLHLS study tried to interview all of the centenarians in the sampled areas.

**Table 4:** Estimates of odds ratios of higher simplified resilience scores (SRS) based on logistic regression.

Dependent variable: higher resilience score	Model 1	Model 2	Model 3	Model 4	Model 5
	Total sample	Total sample	Total sample	Men	Women
*Age group *					
Age 80–89 (ages 65–79)	0.597*** [0.027]	0.740*** [0.036]	0.941 [0.047]	0.837** [0.059]	1.047 [0.076]
Age 90–99 (ages 65–79)	0.443*** [0.020]	0.633*** [0.032]	1.109* [0.062]	0.932 [0.076]	1.276*** [0.099]
Age 100+ (ages 65–79)	0.303*** [0.015]	0.526*** [0.030]	1.261*** [0.083]	1.412*** [0.162]	1.294*** [0.110]
*Demographic* *characteristics*					
Male (Female)		1.341*** [0.053]	1.248*** [0.051]		
Han (Minority)		0.957 [0.073]	0.985 [0.077]	1.15 [0.139]	0.894 [0.091]
Urban (Rural)		1.142*** [0.044]	1.176*** [0.046]	1.241*** [0.078]	1.118** [0.057]
Married (Notmarried including widowed, divorced, and single)		1.523*** [0.066]	1.451*** [0.065]	1.480*** [0.088]	1.400*** [0.096]
Literate (Illiterate)		1.322*** [0.055]	1.206*** [0.052]	1.215*** [0.071]	1.215*** [0.079]
With pension (No pension)		1.529*** [0.080]	1.464*** [0.080]	1.649*** [0.120]	1.255*** [0.110]
*Healthstatus*					
ADL independent (Impaired)			2.145*** [0.106]	2.309*** [0.192]	2.100*** [0.131]
Normal cognition (Impaired)			2.584*** [0.108]	2.344*** [0.153]	2.792*** [0.153]
Whether the province dummy is controlled for	Yes	Yes	Yes	Yes	Yes
Observations	16,428	16,428	16,428	6,941	9,487

Notes: (1) the categories in the parenthesis are reference groups. (2) Standard errors are indicated in the brackets ([ ]). (3) *P < .1; **P < .05; ***P < .01.

**Table 5:** Odds ratios of the impact of the resilience on nonagenarians' likelihood to become a centenarian based on multivariate logistic regressions.

Dependent variable: whether survive to age ≥100	Model 1	Model 2	Model 3	Model 4	Model 5
	Total sample	Total sample	Total sample	Men	Women
Higher resilience (Lower)	1.746*** [0.210]	1.877*** [0.234]	1.431*** [0.190]	1.515* [0.374]	1.404** [0.230]
*Demographic* *characteristic*					
Age—continuous variable		1.492*** [0.097]	1.517*** [0.100]	1.877*** [0.223]	1.405*** [0.116]
Male (Female)		0.703** [0.108]	0.603*** [0.096]		
Han (Minority)		0.670* [0.161]	0.634* [0.156]	0.515 [0.213]	0.664 [0.211]
Urban (Rural)		0.826 [0.108]	0.869 [0.118]	0.675* [0.159]	1.023 [0.176]
Married (Notmarried including widowed, divorced, and single)		1.637** [0.354]	1.588** [0.353]	1.34 [0.355]	3.144** [1.569]
Literate (Illiterate)		1.028 [0.164]	0.949 [0.156]	0.989 [0.222]	0.873 [0.226]
With pension (No pension)		0.964 [0.118]	0.941 [0.118]	0.941 [0.153]	1.005 [0.267]
*Healthstatus*					
ADL independent (Impaired)			1.983*** [0.278]	1.316 [0.338]	2.443*** [0.424]
Normal cognition (impaired)			1.819*** [0.252]	2.961*** [0.744]	1.418** [0.246]
Whether the province dummy is controlled for	Yes	Yes	Yes	Yes	Yes
Whether the year dummy is controlled for	Yes	Yes	Yes	Yes	Yes
Observation	1,528	1,528	1,528	585	943

Notes: the same as in Table 4.
